# GC-biased gene conversion conceals the prediction of the nearly neutral theory in avian genomes

**DOI:** 10.1186/s13059-018-1613-z

**Published:** 2019-01-07

**Authors:** Paulina Bolívar, Laurent Guéguen, Laurent Duret, Hans Ellegren, Carina F. Mugal

**Affiliations:** 1 Department of Ecology and Genetics, Uppsala University, Norbyvägen 18D, 75236 Uppsala, Sweden; 2 Laboratoire de Biologie et Biométrie Évolutive CNRS UMR 5558, Université Claude Bernard Lyon 1, Lyon, France

**Keywords:** Nearly neutral theory, Life-history traits, *d*_*N*_*/d*_*S*_, GC-biased gene conversion, Base composition, Avian genomes

## Abstract

**Background:**

The nearly neutral theory of molecular evolution predicts that the efficacy of natural selection increases with the effective population size. This prediction has been verified by independent observations in diverse taxa, which show that life-history traits are strongly correlated with measures of the efficacy of selection, such as the *d*_*N*_*/d*_*S*_ ratio. Surprisingly, avian taxa are an exception to this theory because correlations between life-history traits and *d*_*N*_*/d*_*S*_ are apparently absent. Here we explore the role of GC-biased gene conversion on estimates of substitution rates as a potential driver of these unexpected observations.

**Results:**

We analyze the relationship between *d*_*N*_*/d*_*S*_ estimated from alignments of 47 avian genomes and several proxies for effective population size. To distinguish the impact of GC-biased gene conversion from selection, we use an approach that accounts for non-stationary base composition and estimate *d*_*N*_*/d*_*S*_ separately for changes affected or unaffected by GC-biased gene conversion. This analysis shows that the impact of GC-biased gene conversion on substitution rates can explain the lack of correlations between life-history traits and *d*_*N*_*/d*_*S*_. Strong correlations between life-history traits and *d*_*N*_*/d*_*S*_ are recovered after accounting for GC-biased gene conversion. The correlations are robust to variation in base composition and genomic location.

**Conclusions:**

Our study shows that gene sequence evolution across a wide range of avian lineages meets the prediction of the nearly neutral theory, the efficacy of selection increases with effective population size. Moreover, our study illustrates that accounting for GC-biased gene conversion is important to correctly estimate the strength of selection.

**Electronic supplementary material:**

The online version of this article (10.1186/s13059-018-1613-z) contains supplementary material, which is available to authorized users.

## Background

With his proposal of the neutral theory of molecular evolution in 1968, Kimura introduced a revolutionizing concept to evolutionary biology, which at that time was strongly influenced by the view that evolution is driven by positive Darwinian selection [1]. Instead, Kimura proposed that at the molecular level deleterious mutations are common, while advantageous mutations are rare, and that in a finite population most evolutionary changes are a consequence of the fixation of neutral mutations due to genetic drift. Kimura thus put a new emphasis on the stochasticity of population genetics, and further established the relationship between sequence conservation and functional importance, which is key to many bioinformatics software for the identification of conserved coding as well as non-coding elements [2].

After realizing the importance of nearly neutral (mainly slightly deleterious) mutations in molecular evolution, Tomoko Ohta extended Kimura’s theory to the nearly neutral theory of molecular evolution [3, 4]. The nearly neutral theory of molecular evolution states that the effectiveness of selection depends on a balance between the strength of random genetic drift and the selection coefficient (*s*) of new mutations [5]. A measure of the strength of genetic drift is the effective population size (*N*_*e*_) [6]. At the population level, aside from the mutation rate, *N*_*e*_ and *s* together determine the level of genetic diversity [7, 8]. Over larger evolutionary time scales, *N*_*e*_ and *s* determine the probability of fixation of new mutations, which ultimately affects the evolutionary rate of change [5, 8]. Therefore, the nearly neutral theory not only forms the basis for a solid null-hypothesis to test for evidence of positive Darwinian selection, but also allows clear predictions to be formulated, which can be tested against data [9]. Specifically, the nearly neutral theory predicts that if *N*_*e*_ is small, the effect of genetic drift is strong, and slightly deleterious mutations are more likely to reach fixation than if *N*_*e*_ is large [5]. Consequently, species with small *N*_*e*_ will accumulate more slightly deleterious substitutions over time than species with large *N*_*e*_. Here, comparison of the evolutionary rate at non-synonymous and synonymous sites allows assessment of the strength of natural selection acting on protein-coding genes, where low effectiveness of selection results in an elevated (but generally < 1), non-synonymous to synonymous substitution rate ratio (*d*_*N*_*/d*_*S*_). Therefore, to test the validity of the nearly neutral theory, the relationship between *N*_*e*_ and *d*_*N*_*/d*_*S*_ can be explored.

Since measuring *N*_*e*_ in natural populations is a challenging task [10–12], a common alternative is to use species characteristics that are associated with *N*_*e*_ as proxies [13–15]. For example, this includes geographic range, where widely distributed species usually have larger *N*_*e*_ than species with a limited geographic range, such as island species [16]; social organization, such as eusociality versus solitarity, where eusocial species usually have lower *N*_*e*_ than solitary species [17]; and mating system, where selfing species usually have lower *N*_*e*_ than outcrossers [18]. It also includes life-history traits that are associated with growth, survival, and reproductive success and strategy [19]. Common examples of life-history traits include, among others, adult body mass or size, maximum longevity, generation time, age of sexual maturity or age at first reproduction, and fecundity. For instance, species with a small body size tend to have a short generation time, large reproductive output, and usually a large *N*_*e*_ [15, 20].

By empirically testing for a relationship between social organization, mating system or life-history traits and *d*_*N*_*/d*_*S*_, several studies have confirmed the prediction of the nearly neutral theory. For example, it has been observed that eusocial insects show higher *d*_*N*_*/d*_*S*_ ratios than solitary species [21]. Another study reported a general trend for higher *d*_*N*_*/d*_*S*_ ratios in the chloroplasts of selfers than of outcrossing species of plants [22]. Moreover, several studies in mammalian taxa have reported strong positive correlations between generation time, body mass, or longevity and *d*_*N*_*/d*_*S*_ in mitochondrial and nuclear genes [23–29]. Similar correlations were also found for non-avian reptiles [30].

However, not all observations are in accordance with the prediction of the nearly neutral theory. An intriguing example is the repeated failure to find a positive correlation between life-history traits and *d*_*N*_*/d*_*S*_ in birds [25, 30, 31]. Since the scenario that the nearly neutral theory does not hold in birds is rather unlikely, there must be a methodological reason or a biological phenomenon that conceals this relationship. To explore the lack of a positive correlation in more detail, the ratio of radical to conservative amino acid substitutions (*K*_*r*_*/K*_*c*_) has been used as an alternative proxy for the efficacy of selection in avian lineages [25, 31]. This revealed a positive correlation between body mass and *K*_*r*_*/K*_*c*_ in birds. This observation questions whether *d*_*N*_*/d*_*S*_ is an appropriate measure for the efficacy of selection in birds. However, the use of the *K*_*r*_*/K*_*c*_ ratio as a measure of the efficacy of selection is not straightforward either given that radical and conservative mutations can be both affected by effectively neutral mutations and hence vary with *N*_*e*_ [30]. Later, Figuet et al. [30] explored the lack of correlations between *d*_*N*_*/d*_*S*_ and life-history traits in birds from another angle. Specifically, they first investigated the hypotheses that life-history traits are poor proxies for *N*_*e*_ and that variation in life-history traits is narrow in birds. They further explored the possibility that a peculiar distribution of fitness effects of new mutations in birds could be responsible for low power to detect a correlation. However, they found evidence to reject all these hypotheses and could thus not explain the lack of a positive correlation. Botero-Castro et al. [32] recently found evidence that sequence alignment quality affects the correlation between life-history traits and *d*_*N*_*/d*_*S*_; after removing putative alignment errors, a positive correlation between *d*_*N*_*/d*_*S*_ and longevity, but not between *d*_*N*_*/d*_*S*_ and body mass, could be recovered. Moreover, they found stronger correlations when previously undetected GC-rich orthologs were included in the analysis. Taken together, observations from previous studies suggest that *d*_*N*_*/d*_*S*_ might not be an appropriate measure for the efficacy of selection in birds, potentially because there is some force that biases estimates of *d*_*N*_*/d*_*S*_ and thereby obscures the correlation between *d*_*N*_*/d*_*S*_ and life-history traits in birds.

It is well documented that avian genomes are strongly impacted by GC-biased gene conversion (gBGC) [33–36]. gBGC is a process associated with meiotic recombination that leads to the preferential fixation of GC (strong: S) over AT (weak: W) alleles in GC/AT heterozygous sites close to recombination-initiating double-strand breaks [37, 38]. It acts in a manner similar to directional selection, increasing the probability of fixation of S over W alleles [39]. Thus, like the strength of natural selection, also the strength of gBGC increases with *N*_*e*_ and recombination rate, which both vary along the genome and among species. gBGC can therefore interfere with natural selection and bias inferences of the strength and efficacy of selection based on divergence and/or diversity data [40–47]. The net impact of gBGC on substitution rates also depends on the relative contribution of substitutions from AT to GC (W-to-S) and from GC to AT (S-to-W), which is reflected in ΔGC, the difference between the equilibrium GC content (GC*) and the ancestral GC content [41, 48]. If ΔGC differs between synonymous and non-synonymous substitutions, gBGC may increase or decrease estimates of *d*_*N*_*/d*_*S*_ depending on the relative impact of gBGC on synonymous and non-synonymous substitution rates.

Here, we revisit the relationship between *d*_*N*_*/d*_*S*_ and life-history traits in birds, and investigate if the impact of gBGC on *d*_*N*_*/d*_*S*_ conceals the correlation between life-history traits and *d*_*N*_*/d*_*S*_ in the avian clade. In order to distinguish the impact of gBGC on lineage-specific substitution rates from the impact of selection, we estimate *d*_*N*_*/d*_*S*_ separately for different substitution categories, namely W-to-S and S-to-W changes, which are both affected by gBGC, and GC-conservative changes, i.e., S-to-S and W-to-W, which are not affected by gBGC. To do so, we adapt a recently implemented approach that explicitly allows for non-stationary base composition [49]. This analysis provides clear evidence that gBGC conceals the correlation between life-history traits and *d*_*N*_*/d*_*S*_ in birds. Moreover, it stresses the importance of accounting for gBGC to correctly estimate the strength of selection in comparative genomics studies, which seems particularly important when the impact of gBGC varies among lineages. Finally, we propose a new statistic, *d*_*N*_*/d*_*S*_ based on GC-conservative changes, together with a program to perform estimates of the strength of selection after accounting for the impact of gBGC.

## Results

### gBGC conceals the correlation between dN/dS and life-history traits in birds

We explored the relationship between estimates of *d*_*N*_*/d*_*S*_ based on publicly available coding sequence alignments of 7986 genes in 47 avian species and three life-history traits (body mass, longevity, and age of sexual maturity). To estimate non-synonymous and synonymous substitution rates, we applied methods implemented in the bio++ libraries [50, 51]. First, we fitted a non-stationary homogeneous YN98 substitution model by maximum likelihood, to retrieve the most likely branch lengths, codon frequencies at the root, and model parameters. Second, we applied a recently developed approach based on substitution mapping to estimate *d*_*N*_*/d*_*S*_ [49]. We further modified this approach to split non-synonymous (and synonymous) counts in three categories (W-to-S, S-to-W, and GC-conservative changes, i.e., S-to-S plus W-to-W), such that *d*_*N*_*/d*_*S*_ was estimated separately for each of those categories. Briefly, for each of these categories, we used substitution mapping [52] to estimate the expected number of substitutions on each branch given the distribution of the scenarios provided by the previously optimized model and tree. We normalized these counts by the expected numbers of such substitutions for the same scenarios under a neutral model (i.e., *ω* = 1) [49] (see Methods section).

**Fig. 1:**
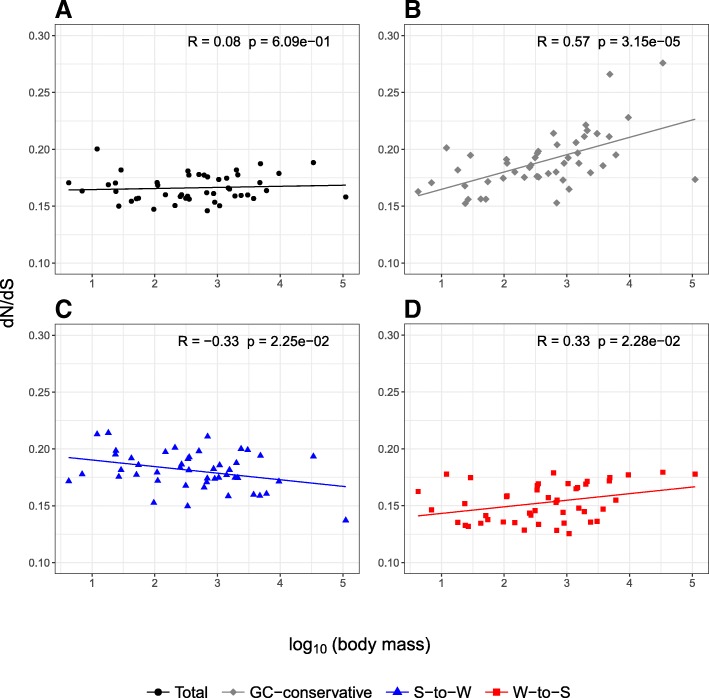
Relationship between *d*_*N*_*/d*_*S*_ and log-transformed body mass for different substitution categories. **a** All substitution categories together (total; black circles), **b** GC-conservative substitutions (gray diamonds), **c** S-to-W substitutions (blue triangles), and **d** W-to-S substitutions (red squares). Each dot corresponds to a terminal branch of the avian phylogeny. Regression lines for each substitution category are shown using the same color scheme. Pearson correlation coefficients and their statistical significance are provided in the right upper corner of each panel

Estimates of *d*_*N*_*/d*_*S*_ based on all substitutions together were on average smaller than estimates based on only GC-conservative changes, but differences between *d*_*N*_*/d*_*S*_ based on all substitutions and *d*_*N*_*/d*_*S*_ based on GC-conservative changes ranged from negative to positive values (Additional file 1: Table S1). Data for GC-conservative changes showed very strong positive correlations between *d*_*N*_*/d*_*S*_ and all life-history traits, most prominently for body mass followed by the age of sexual maturity and longevity (Table 1). In contrast, none of the relationships between *d*_*N*_*/d*_*S*_ based on all substitutions together and life-history traits were statistically significant (Fig. 1 and Table 1). Data for the two substitution categories influenced by gBGC showed weak correlations with opposite directions to each other. Since correlation analysis can be affected by phylogenetic relationships of species and their life-history traits, we further performed correlation analysis after accounting for the phylogenetic relationships of species. The conclusion remained the same after accounting for the phylogenetic relationships of species when estimating the correlation between *d*_*N*_*/d*_*S*_ and body mass (Additional file 2: Table S2). For the other two life-history traits, longevity and age of sexual maturity, correlations for *d*_*N*_*/d*_*S*_ based on GC-conservative changes lost their statistical significance, but were still larger than correlations for *d*_*N*_*/d*_*S*_ based on all substitution categories. Note that correction for phylogenetic relationships is the correct practice to perform correlation analysis among related species. Here, uncorrected correlations are reported in the main text and corrected ones in the Supplement in order to allow for a more direct comparison between this and previous studies.

**Table 1:** Pearson correlation coefficients (*R*) and their statistical significance between *d*_*N*_*/d*_*S*_ for different substitution categories and different proxies for *N*_*e*_. Significant correlations are highlighted in italics

Life history trait	Total		GC-conservative		S-to-W		W-to-S	
	*R*	*p* value	*R*	*p* value	*R*	*p* value	*R*	*p* value
Body mass	0.08	6.09 *×* 10^−1^	*0.57*	*3.15 × 10* ^*−5*^	*−0.33*	*2.25 × 10* ^*−2*^	*0.33*	*2.28 × 10* ^*−2*^
Longevity	0.08	6.25 *×* 10^−1^	*0.32*	*4.63 × 10* ^*−2*^	−0.22	1.64 *×* 10^−1^	0.27	9.51 *×* 10^−2^
Age of sexual maturity	0.08	5.85 *×* 10^−1^	*0.45*	*1.97 × 10* ^*−3*^	−0.19	1.99 *×* 10^−1^	0.28	6.41 *×* 10^−2^

S-to-W: strong to weak, W-to-S: weak to strong

To test the robustness of the results obtained from substitution mapping, we repeated the analyses using two other substitution models implemented in the bio++ libraries, model T92X3 and model L95X3 (for details see Methods). Estimates of *d*_*N*_*/d*_*S*_ and consequently the strength of the correlations with life-history traits were highly similar to that obtained using the YN98 model (Additional file 2: Figures S1 and S2). This suggests that our inferences are robust to the underlying codon substitution model.

Estimates of substitution rates have been shown to be biased for genes that have gene trees discordant to the species tree if substitution rates are estimated based on the species tree [53]. To examine the possible influence of gene tree discordance on our observations, we repeated the analysis using a gene-by-gene approach, where the number of non-synonymous and synonymous substitutions and sites were estimated for each gene separately with their respective gene tree. In order to calculate average species-specific substitution rates for the different substitution categories, we divided the sum of the substitution counts over all genes of the respective category by the sum of the expected number of substitutions over all genes of the same category. Additional file 2: Tables S3 and S4 show that the lack of a positive correlation between life-history traits and *d*_*N*_*/d*_*S*_ based on all substitutions as well as the presence of a significant positive correlation between life-history traits and *d*_*N*_*/d*_*S*_ based on GC-conservative changes are robust to gene tree heterogeneity. However, compared to the analysis based on the species tree, correlations between life-history traits and *d*_*N*_*/d*_*S*_ based on W-to-S substitutions lost significance for the gene-by-gene approach.

### The impact of gBGC on *d*_*N*_/*d*_*S*_ varies among lineages

**Fig. 2:**
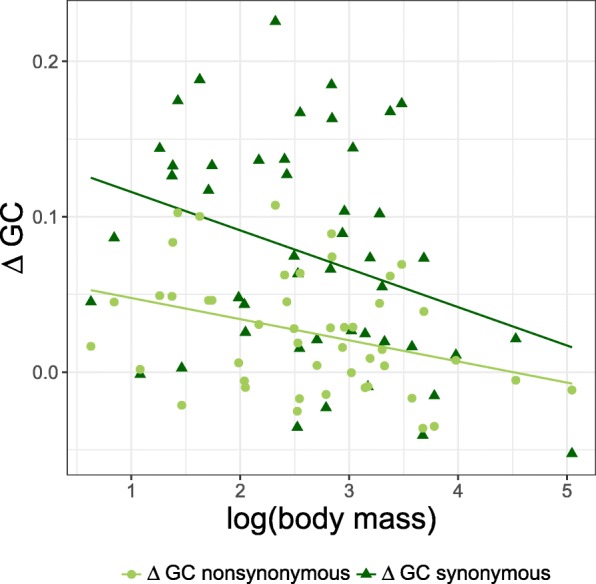
Relationship between ΔGC and body mass. ΔGC non-synonymous (light green circles) and ΔGC synonymous (dark green triangles). Regression lines for each substitution category are shown using the same color scheme

The overall impact of gBGC on total substitution rates (and consequently on *d*_*N*_*/d*_*S*_ based on all substitution categories) not only depends on the strength of gBGC but also on the relative contribution of S-to-W and W-to-S substitutions to the total number of substitutions. While the strength of gBGC is reflected in the equilibrium GC content (GC*) [36, 54], the relative contribution of S-to-W and W-to-S to the total number of substitutions in a certain lineage is governed by its ancestral GC content (i.e., the GC content at its most recent ancestral node). For each lineage, we computed the difference between the equilibrium GC content of the substitution process (GC*) and the ancestral GC content (denoted as ΔGC) (see Methods) to summarize the dynamics of base composition, separately for synonymous and non-synonymous substitutions. We observed that ΔGC differed between synonymous and non-synonymous substitution rates and varied among lineages. Interestingly, in the majority of the avian lineages analyzed, ΔGC synonymous was larger than ΔGC non-synonymous (mean ± standard deviation; ΔGC synonymous = 0.076 ± 0.071 and ΔGC non-synonymous = 0.026 ± 0.038), which indicates that in avian lineages neutrally evolving sites are on average further away from their equilibrium GC content than sites evolving under selection. This would suggest that on average the impact of gBGC is lower on *d*_*N*_ than on *d*_*S*_, which will influence the net impact of gBGC on the *d*_*N*_*/d*_*S*_ ratio [41, 48] and could explain why *d*_*N*_*/d*_*S*_ based on all substitution categories together is on average decreased (compared to *d*_*N*_*/d*_*S*_ based on GC-conservative changes). ΔGC synonymous and ΔGC non-synonymous showed a negative relationship with body mass (Fig. 2; ΔGC synonymous: *R* = − 0.33, *p* value = 2.36 × 10^− 2^; and ΔGC non-synonymous: *R* = − 0.34, *p* value = 1.81 × 10^− 2^), indicating that species with large *N*_*e*_ and thus higher strength of gBGC generally are further away from their equilibrium than species with small *N*_*e*_.

### Comparison with and re-analysis of previously investigated bird datasets

Several previous studies have addressed the relationship between *d*_*N*_*/d*_*S*_ and life-history traits in birds [25, 30–32]. Table 2 provides an overview of the relationship between *d*_*N*_*/d*_*S*_ and body mass among studies (including our study), which illustrates the consistent lack of a significant positive correlation if gBGC is not accounted for. Although all these studies, like us, used a substitution mapping approach to estimate branch-specific *d*_*N*_*/d*_*S*_, our study differs from previous work in three key aspects.

**Table 2:** Description and Pearson correlation coefficients (*R*) and their statistical significance between *d*_*N*_*/d*_*S*_ (based on all substitution categories together) and body mass reported in different studies. Significant correlations are highlighted in italics

Study (dataset)	Number of genes	Description	*Neornithes*		*Neoaves*	
			*R*	*p* value	*R*	*p* value
Weber et al. [31]	921	Alignments from Jarvis et al. [57] present in all 48 species	*− 0.43*	*2.70 × 10* ^*− 3*^	NA	NA
Figuet et al. [30]	1077	Alignments from Jarvis et al. [57] for orthologs that are also present in mammals and non-avian sauropsids. Max missing data of 6 species	0.13	3.20 *×* 10^− 1^	NA	NA
Botero-Castro et al. [32] (Figuet+HMMclean)	1077	Dataset from Figuet et al. 2016 + alignment filtering based on HMMclean	0.17	2.80 *×* 10^− 1^	*0.42*	*4.00 × 10* ^*− 3*^
Botero-Castro et al. [32] (Botero-Castro)	1077 + 1245	Figuet+HMMclean dataset + 1245 previously non-annotated GC-rich genes present in at least 10 species	0.23	1.30 *×* 10^− 1^	*0.59*	*7.34 × 10* ^*−5*^
This study	7986	Alignments from Jarvis et al. [57] Max missing data of 6 species	0.08	6.09 *×* 10^−1^	0.19	2.19 *×* 10^−1^

First, the underlying substitution model that we used explicitly allows for non-stationary base composition, in contrast to the assumption of stationary base composition used in previous studies. Second, the subset of genes analyzed and the alignment procedures varied between studies (Table 2). Finally, only in our study, *d*_*N*_*/d*_*S*_ was separately estimated for S-to-W, W-to-S, and GC-conservative substitution categories. Comparison of the relationships between *d*_*N*_*/d*_*S*_ and life-history traits among studies is therefore limited to estimates of *d*_*N*_*/d*_*S*_ based on all substitutions together (Table 2).

In order to improve comparability between results and to be able to distinguish between the effects of the underlying methodological differences (related to the stationarity assumption) and gene dataset, we re-analyzed two datasets with our revised approach, the cleaned alignments of Figuet et al. [30] (referred to as Figuet+HMMclean) and the alignments of Botero-Castro et al. [32]. Note that these two datasets are subsets of the avian dataset published by Jarvis et al. [57]. We addressed two different aspects when re-analyzing these two data sets. First, we applied our modified (non-stationary) model to analyze all substitution categories together. So, instead of a method that assumes stationarity in base composition as implemented in the original publications, we used a method that allows for non-stationarity. Second, we re-analyzed previous datasets also using the modified (non-stationary) model but this time limited to only GC-conservative changes in order to investigate the impact of gBGC.

Similar to the original publications, correlations between life-history traits and *d*_*N*_*/d*_*S*_ based on all substitution categories together (which are affected by gBGC) were only marginally or non-significant (Table 3). This suggests that the relaxation of the stationarity assumption has only a minor impact on the results. On the other hand, correlations between life-history traits and *d*_*N*_*/d*_*S*_ based on GC-conservative changes only (which are not affected by gBGC), were strong and significant for all datasets, which suggests that gBGC has a major impact on the results regardless of the data set analyzed. In addition, as observed in the original studies, Paleognathae (ratites and tinamous) and Galloanserae (gamefowl and waterfowl) appeared to be outliers in the Figuet+HMMclean and the Botero-Castro et al. datasets (Additional file 2: Figures S3-S5); estimates of *d*_*N*_*/d*_*S*_ were lower than expected by their respective body mass. We therefore followed Botero-Castro et al. [32] and repeated the correlation analysis within the group of Neoaves only. Again in agreement with previous observations [32], correlations between *d*_*N*_*/d*_*S*_ and life-history traits were stronger within Neoaves than within Neornithes (Table 3 and Additional file 2: Figures S3-S5,). However, after accounting for the phylogenetic relationships of species, the difference in the strength of correlations within Neoaves and within Neornithes reduced to only a minor effect (Additional file 2: Table S5). This suggests that correction for phylogenetic relationships of species is important and indicates that contrary to previous suggestions [32], life-history traits do not seem to lose power to properly describe variation in *N*_*e*_ over large evolutionary distances. Nevertheless, our results provide evidence that gBGC weakens the correlation between *d*_*N*_*/d*_*S*_ and life-history traits in birds and that this observation is robust to the underlying gene dataset.

**Table 3:** Pearson correlation coefficients (*R*) and their statistical significance between *d*_*N*_*/d*_*S*_ and body mass for different datasets re-analyzed in the present study. Significant correlations are highlighted in italics

Dataset	*Neornithes*		*Neoaves*		*Neornithes* GC-conservative		*Neoaves* GC-conservative	
	*R*	*p* value	*R*	*p* value	*R*	*p* value	*R*	*p* value
Figuet+HMMclean	0.11	4.66 *×* 10^−1^	0.3	6.00 *×* 10^−1^	*0.51*	*3.53 × 10* ^*−4*^	*0.75*	*3.68 × 10* ^*−8*^
Botero-Castro	*0.33*	*2.68 × 10* ^*−2*^	*0.69*	*1.41 × 10* ^*−6*^	*0.55*	*1.23 × 10* ^*−4*^	*0.83*	*5.86 × 10* ^*−11*^
Original dataset of our study	0.08	6.09 *×* 10^−1^	0.19	2.19 *×* 10^−1^	*0.57*	*3.15 × 10* ^*−5*^	*0.72*	*7.34 × 10* ^*−8*^

### Local GC content and chromosome size affect estimates of *d*_*N*_*/d*_*S*_ but not its correlations with life-history traits

**Fig. 3:**
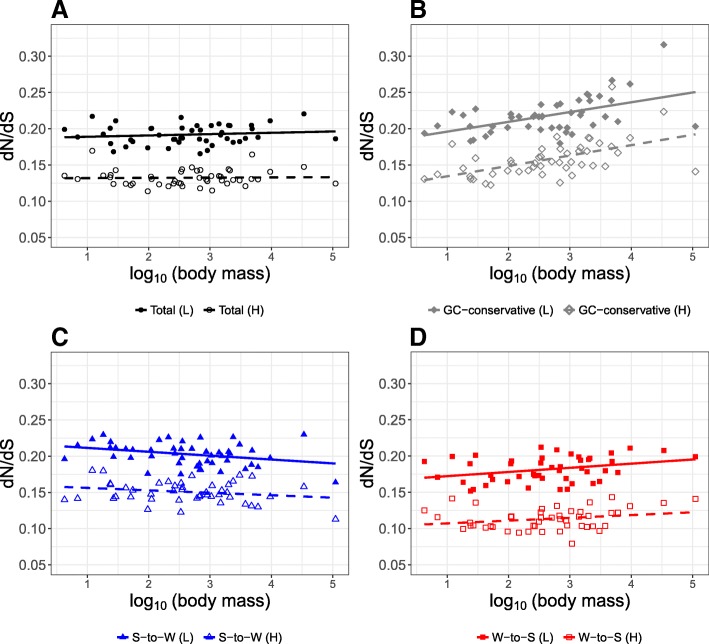
Relationships between *d*_*N*_*/d*_*S*_ and body mass separately for GC-poor genes (low GC content) and GC-rich genes (high GC content). Low GC content (L; filled shapes) and high GC content (H; hollow shapes). **a** All substitution categories together (total; black circles), **b** GC-conservative substitutions (gray diamonds), **c** S-to-W substitutions (blue triangles), and **d** W-to-S substitutions (red squares). Each dot corresponds to a terminal branch of the avian phylogeny. Regression lines for each substitution category are shown using the same color scheme. Solid lines correspond to GC-poor genes, dashed lines to GC-rich genes

Previous studies have observed differences in estimates of *d*_*N*_*/d*_*S*_ between GC-rich and GC-poor genes [32]. In order to test if our results are robust to local variation in GC content, we split the dataset into two groups according to their GC content, i.e., high GC content and low GC content. We then estimated *d*_*N*_*/d*_*S*_ separately for the two datasets and investigated the correlation between body mass and *d*_*N*_*/d*_*S*_ for different substitution categories. The correlation between body mass and *d*_*N*_*/d*_*S*_ based on GC-conservative changes was significantly positive both for GC-rich and GC-poor genes, but not significant when analyzing all substitutions together. Interestingly, correlations were similar between datasets, but estimates of *d*_*N*_*/d*_*S*_ were consistently lower for GC-rich genes than for GC-poor genes, irrespective of the substitution category (Fig. 3, Table 4, and Additional file 2: Table S5, for correlations after correction for phylogenetic relationships of species). The difference in *d*_*N*_*/d*_*S*_ between GC-rich and GC-poor genes appears to be independent of the effect of gBGC on *d*_*N*_*/d*_*S*_, since all substitution categories were affected in the same way. Instead, the difference in *d*_*N*_*/d*_*S*_ seems to be a consequence of both higher *d*_*S*_ and lower *d*_*N*_ in GC-rich genes as compared to GC-poor genes (relative difference in *d*_*S*_ and *d*_*N*_ for GC-conservative changes, respectively, significance based on a *t*-test; Δ*d*_*S*_ = 12.57, *p* value < 2.2 × 10^− 16^, Δ*d*_*N*_ = − 6.51, *p* value = 4.89 × 10^− 8^; for all substitution categories together, Δ*d*_*S*_ = 31.02, *p* value < 2.22 × 10^− 16^, Δ*d*_*N*_ = − 13.41, *p* value < 2.22 × 10^− 16^; Additional file 2: Figure S6).

**Table 4:** Pearson correlation coefficients and their statistical significance between *d*_*N*_*/d*_*S*_ for different substitution categories and body mass for genes with low and high GC content, and for genes located in microchromosomes or macrochromosomes. Significant correlations are highlighted in italics

	Total		GC-conservative		S-to-W		W-to-S	
	*R*	*p* value	*R*	*p* value	*R*	*p* value	*R*	*p* value
High GC content	0.03	8.54 *×* 10^−1^	*0.53*	*1.22 × 10* ^*−4*^	− 0.21	1.53 *×* 10^−1^	0.25	9.43 *×* 10^−2^
Low GC content	0.13	3.83 *×* 10^−1^	*0.52*	*1.93 × 10* ^*−4*^	*− 0.32*	*2.73 × 10* ^*−2*^	*0.31*	*3.16 × 10* ^*−2*^
Microchromosomes	0.14	3.45 *×* 10^−1^	*0.55*	*7.49 × 10* ^*−5*^	− 0.26	7.79 *×* 10^−2^	*0.40*	*5.42 × 10* ^*−3*^
Macrochromosomes	0.01	9.27 *×* 10^−1^	*0.47*	*7.65 × 10* ^*−4*^	− 0.27	6.18 *×* 10^−2^	0.17	2.61 *×* 10^−1^

S-to-W: strong to weak, W-to-S: weak to strong

To further elaborate on the relationship between local GC content and estimates of *d*_*N*_*/d*_*S*_, we explored the association between chromosome size and estimates of *d*_*N*_*/d*_*S*_. Avian genomes are characterized by a very large variation in chromosome size, with macrochromosomes (here defined as chromosomes larger than 100 Mb) having lower overall recombination rate and lower GC content than microchromosomes (here smaller than 16 Mb). Hence, GC-rich genes are more often found on microchromosomes. Consistent with what has been described above, we observed lower *d*_*N*_*/d*_*S*_ estimates for microchromosomes than for macrochromosomes (Table 4 and Additional file 2: Figure S7). Also similar to above, the difference in *d*_*N*_*/d*_*S*_ seemed to be a consequence of both higher *d*_*S*_ and lower *d*_*N*_ in microchromosomes than in macrochromosomes (relative difference in *d*_*S*_ and *d*_*N*_ for GC-conservative changes, respectively, significance based on a *t*-test; Δ*d*_*S*_ = 6.83, *p* value = 1.64 × 10^− 8^, Δ*d*_*N*_ = − 8.91, *p* value = 1.42 × 10^− 11^; for all substitution categories together, Δ*d*_*S*_ = 11.15, *p* value = 1.14 × 10^− 14^, Δ*d*_*N*_ = − 10.62, *p* value = 5.75 × 10^− 14^; Additional file 2: Figure S8). Taken together, these results indicate that local GC content and chromosome size affect estimates of *d*_*N*_*/d*_*S*_. Since GC content and chromosome size are correlated, it is difficult to know to what extent the effect of chromosome size simply is an effect of GC content, or vice versa. The correlation between life-history traits and *d*_*N*_*/d*_*S*_ based on GC-conservative changes is on the other hand robust to variation in local GC content and chromosome size.

## Discussion

The lack of a positive relationship between *d*_*N*_*/d*_*S*_ and life-history traits in avian taxa has been puzzling and has therefore been explored from various angles [25, 30–32]. Our results offer a solution to the puzzle by providing clear evidence that gBGC distorts *d*_*N*_*/d*_*S*_ such that the ratio does not reflect the variation in the efficacy of selection among avian lineages. Strong positive correlations were recovered after accounting for gBGC. In contrast, and in agreement with earlier observations, life-history traits and *d*_*N*_*/d*_*S*_ were not positively correlated when the estimation of *d*_*N*_*/d*_*S*_ was based on all substitution categories together without accounting for gBGC. Thus, our study clearly illustrates that accounting for gBGC is crucial to correctly estimate the strength of selection in comparative genomic studies across taxa that are affected by gBGC.

gBGC has been observed in a wide range of taxa [38]. Both theoretical investigations and empirical observations suggest that gBGC shows a strong impact on *d*_*N*_*/d*_*S*_ [41, 42, 45, 46, 48]. Interestingly, while gBGC was found to increase estimates of *d*_*N*_*/d*_*S*_ in mammals and fishes [55, 56], previous work in birds (chicken and flycatchers) suggested that *d*_*N*_*/d*_*S*_ was decreased by gBGC [41, 55]. However, our avian analyses indicate a more nuanced picture. While we observe that the impact of gBGC in the majority of avian lineages indeed results in an underestimation of *d*_*N*_*/d*_*S*_, in some it results in an overestimation (Fig. 1, Additional file 1: Table S1). The net effect of gBGC on *d*_*N*_*/d*_*S*_ depends on *N*_*e*_ and recombination rate, but also builds upon the relative contribution of W-to-S and S-to-W substitutions to synonymous versus non-synonymous substitution rates [41]. All these parameters are reflected in the dynamics of base composition. Since we found that the dynamics of base composition differs between neutrally evolving and selected sites, and varies substantially among lineages, this might very well explain why gBGC shows contrasting effects on *d*_*N*_*/d*_*S*_. However, more specific models are needed to fully understand how gBGC alters *d*_*N*_*/d*_*S*_.

Besides gBGC, deviations of individual gene trees from the species tree as a consequence of incomplete lineage sorting (ILS) could impact estimates of *d*_*N*_*/d*_*S*_ if estimation is based on the species tree [53]. Specifically, it has been suggested that gene tree discordance may lead to artificially higher substitution rates and also to an increase in *d*_*N*_*/d*_*S*_, especially when levels of ILS are high. Since ILS has been suggested to be abundant in birds [57], it is possible that gene tree heterogeneity could contribute to the observed correlations between life-history traits and *d*_*N*_*/d*_*S*_ if the estimation of *d*_*N*_*/d*_*S*_ is based on the species tree. On the other hand, it has been suggested that gBGC increases the error rate in tree inference [29, 34]. As a consequence, the estimation of *d*_*N*_*/d*_*S*_ based on individual gene trees could be affected by a high error rate in tree inference, particularly so for genes located in regions strongly affected by gBGC. However, comparison of correlations between life-history traits and *d*_*N*_*/d*_*S*_, one based on the species tree and one based on individual gene trees, suggests that our conclusion is robust to gene tree heterogeneity. For both approaches, we observe no significant correlations between life-history traits and *d*_*N*_*/d*_*S*_ based on all substitution categories, but significant positive correlations if *d*_*N*_*/d*_*S*_ is based on GC-conservative changes only (Fig. 1, Table 1, and Additional file 2: Table S3).

Yet other factors that could impact estimates of *d*_*N*_*/d*_*S*_ and consequently the correlation between life-history traits and *d*_*N*_*/d*_*S*_ in birds are alignment quality and/or missing genes [32]. After accounting for alignment errors (and inclusion of previously undetected genes), Botero-Castro et al. [32] found a significant positive correlation between *d*_*N*_*/d*_*S*_ and longevity. They also found that *d*_*N*_*/d*_*S*_ was significantly correlated with body mass in the Neoaves clade, i.e., all birds except Paleognathae (ratites and tinamous) and Galloanserae (gamefowl and waterfowl). The authors suggested that the latter group of birds might be outliers, since for those birds current life-history traits might not properly reflect long-term *N*_*e*_ (see also ref. [25]). However, after correction for phylogenetic relationships of species, we no longer observe striking differences between correlations for their dataset. Moreover, in the larger dataset analyzed in this study, we do not observe that Paleognathae and Galloanserae deviate from the overall pattern. Taken together, the hypothesis that gBGC conceals the prediction of the nearly neutral theory appears robust to the set of genes analyzed as well as to alignment quality. *d*_*N*_*/d*_*S*_ based on GC-conservative changes showed strong positive correlations with all life-history traits analyzed in this study in all datasets, while correlations were weak when analyzing all substitutions together. This demonstrates that gene set and alignment quality alone do not explain the lack of a positive correlation between life-history traits and *d*_*N*_*/d*_*S*_ in birds.

The correlations between life-history traits and *d*_*N*_*/d*_*S*_ based on GC-conservative changes were robust to variation in local GC content and chromosome location. We observed significant positive correlations for GC-rich and GC-poor genes, as well as for genes located in macrochromosomes or in microchromosomes. Estimates of *d*_*N*_*/d*_*S*_ were lower for genes with higher GC content and for genes located in microchromosomes. This reduction appears to be unrelated to gBGC, as it is observed for all substitution categories including GC-conservative substitutions. We found that the reduction in *d*_*N*_*/d*_*S*_ is a result of lower *d*_*N*_ values at the same time as *d*_*S*_ values are increased. Higher *d*_*S*_ values for microchromosomes than for macrochromosomes have been reported before and were suggested to be a result of higher mutation rates on microchromosomes than on macrochromosomes [58]. Still, *d*_*N*_ was reduced for GC-rich genes and genes located on microchromosomes. In birds, recombination rate is positively correlated with GC content and negatively with chromosome size [59, 60]. Thus, the reduction in *d*_*N*_ could be a result of a lower interference between selected sites (Hill-Robertson interference) due to higher recombination rates [61]. Alternatively, more rapid sequence saturation in GC-rich regions relative to GC-poor regions could influence estimation of *d*_*N*_*/d*_*S*_. Weber et al. [31] investigated this particular concern in avian genomes and found that sequence saturation affects synonymous sites more strongly than non-synonymous sites, leading to a greater underestimation of *d*_*S*_ relative to *d*_*N*_ [31]. As a consequence, the *d*_*N*_*/d*_*S*_ ratio would be increased by sequence saturation in GC-rich regions relative to GC-poor regions. Given that we observe lower *d*_*N*_*/d*_*S*_ in GC-rich regions than in GC-poor regions, we believe that sequence saturation should not be of concern for our conclusions.

## Conclusions

We have explored the role of gBGC behind the apparent lack of a positive correlation between life-history traits and *d*_*N*_*/d*_*S*_ in birds, a correlation that would be expected based on the prediction of the nearly neutral theory of molecular evolution. By estimating nucleotide substitution rates separately for different substitution categories, we observe strong positive correlations between three life-history traits (body mass, age of sexual maturity and longevity) and *d*_*N*_*/d*_*S*_ when the estimation of *d*_*N*_*/d*_*S*_ is based on GC-conservative substitutions only. No significant correlations were observed when estimation of *d*_*N*_*/d*_*S*_ was based on all substitutions together, which can be ascribed to the influence of gBGC on S-to-W or W-to-S substitutions. gBGC thus impacts the correlation between life-history traits and *d*_*N*_*/d*_*S*_, and after accounting for gBGC, we find that the efficacy of selection increases with proxies for *N*_*e*_, as predicted by the nearly neutral theory. The impact of gBGC on *d*_*N*_*/d*_*S*_ varies substantially among lineages, where it increases the *d*_*N*_*/d*_*S*_ ratio in some lineages, but decreases it in others. Our analysis suggests that these contrasting effects are related to differences in the dynamics of base composition between non-synonymous and synonymous substitutions. Moreover, altogether our study clearly illustrates that gBGC interferes with natural selection and that accounting for gBGC is a crucial step to correctly infer measures of the efficacy of natural selection such as the *d*_*N*_*/d*_*S*_ ratio in comparative genomic studies. In light of this observation, we suggest that conclusions of previous studies that ignored the impact of gBGC on molecular evolutionary rates might need a careful re-evaluation. We here provide a protocol to do this.

## Methods

### Multiple sequence alignments

We downloaded publicly available coding sequence alignments of 8253 orthologous genes from 48 avian genomes and their inferred phylogenetic tree from Jarvis et al. [57]. We excluded the white-tailed eagle (*Haliaeetus albicilla*), since the branch length between this species and another eagle species included in the data set (bald eagle, *Haliaeetus leucocephalus*), was too short to reliably estimate *d*_*N*_*/d*_*S*_ ratios [62]. We further excluded Z-linked genes according to the chicken annotation from Ensembl v90 (Gallus_gallus-5.0) [63, 64], since the efficacy of selection differs between sex-linked and autosomal genes [65, 66]. This resulted in a dataset of 7986 orthologous genes of 47 bird species. In addition, following previous approaches [30], only codons represented in at least 41 species were retained.

Two additional sets of multiple sequence alignments were retrieved from Botero-Castro et al. [32]. The first of these, which we refer to as the Figuet+HMMclean dataset, is a set of 1077 avian genes analyzed by Figuet et al. [30]. This dataset is a subset of the Jarvis et al. [57] data, which are used in the present study. The dataset consisted of genes shared by sauropsids and mammals based on orthology prediction between chicken, green anole, and human. Misaligned sites in these alignments were subsequently filtered using HMMclean [32]. The second set of alignments, which we refer to as Botero-Castro dataset, included all genes in the Figuet+HMMclean dataset plus a set of 1245 previously undetected avian GC-rich genes [32]. For further description on the gene sets and cleaning methods, we refer to the original publications.

### Concatenation of sequence alignments

Concatenation of gene-sequence alignments into a single alignment caused problems with large computational memory for the subsequent estimation of substitution rates.

Therefore, we randomly concatenated gene alignments into 20 different bins of roughly 400 genes each and estimated substitution rates for each bin. The average *d*_*N*_ and *d*_*S*_ across all bins were computed to obtain genome-wide averages of *d*_*N*_*/d*_*S*_. Concatenated alignments were used if estimation of substitution rates was based on the species tree. This approach was used to increase the signal-to-noise ratio for individual estimates. We repeated the analyses for 20 bins grouped according to their GC content. To do so, we first calculated the average GC content per species for each gene. Then, we calculated the mean GC content per gene over all species and ranked the genes accordingly. In order to separate genes into two classes of GC-rich and GC-poor genes, we split the 20 bins into two groups, each containing 10 bins with the highest and lowest GC content, respectively. We further repeated the analyses by binning genes according to their genomic location; one bin of genes located in microchromosomes and one bin of genes located in macrochromosomes, respectively. To avoid analyzing genes located in intermediate size chromosomes, we included genes located in chromosomes smaller than 16 Mb (chromosomes 14–28; a total of 1369 genes) in the category of microchromosomes and genes located in chromosomes larger than 100 Mb (chromosomes 1–3; a total of 1321 genes) in the category of macrochromosomes. We excluded genes from chromosome 1 if their location in the zebra finch was chromosome 1A, representing a well-known chromosomal rearrangement, or unknown (according to Ensemble v.90; [64]).

For the Figuet+HMM and Botero-Castro datasets, the same binning procedure as for the main analyses was performed. We randomly concatenated gene alignments into bins of roughly 400 genes each and estimated substitution rates for each bin. Subsequently, the average *d*_*N*_ and *d*_*S*_ estimates across all bins were computed to obtain genome-wide averages of *d*_*N*_*/d*_*S*_.

### Estimation of lineage-specific *d*_*N*_/*d*_*S*_

We estimated *d*_*N*_*/d*_*S*_ for each bin by using the bio++ libraries [50, 51] and the total evidence nucleotide tree (TENT) species tree from Jarvis et al. [57]. As a first step, we used a non-stationary homogeneous codon model of molecular evolution implemented in bppml to retrieve the most likely branch lengths, codon frequencies at the root, and substitution model parameters. We implemented three different substitution models, which all allow for different GC content dynamics between codon positions, YN98 (F3X4) [67], T92X3 [68], and L95X3 [69]. As a second step, we used MapNH for substitution mapping to estimate *d*_*N*_ and *d*_*S*_, and then *d*_*N*_*/d*_*S*_ [49, 52, 70]. The idea of the second step is to compute, on any branch, the expectation of any random variable over all the possible histories provided by the model and tree optimized in the first step. For example, *d*_*S*_ is the ratio of the number of synonymous substitutions performed by the model divided by the number of synonymous substitutions that would have been performed by a similar model, but set as neutral (i.e., *ω* = 1 for YN98 model). So, the program computes the expectation of both numbers and returns the ratio as *d*_*S*_. In an analogous way, *d*_*N*_ is computed for non-synonymous substitutions. Since it is possible to consider any kind of event, we also computed the ratios of the expected numbers of non-synonymous and synonymous substitutions restricted to specific substitution categories, such as S-to-S, S-to-W, W-to-S, and W-to-W. For example, *d*_*S*_ for the W-to-S substitution category is the expected number of W-to-S synonymous substitutions, divided the number of such substitutions that would have been performed by a similar neutral model. To combine S-to-S and W-to-W substitution rates into one category, i.e., the GC-conservative category, we weighed the S-to-S and W-to-W substitution rates of each species according to the GC content at the most recent internal node of each particular tip of the tree. To reconstruct the most likely ancestral sequence at each node, we used the bppancestor program [51, 71].

To account for gene tree heterogeneity, we estimated *d*_*N*_*/d*_*S*_ with an additional approach, where the number of substitutions was estimated separately for each gene (instead of concatenated bins) using gene-specific phylogenetic trees (based on the first and second codon positions) from Jarvis et al. [57]. In order to obtain genome-wide averages of *d*_*N*_, *d*_*S*_, and *d*_*N*_*/d*_*S*_, the sum of number of substitutions for each substitution category over all genes was computed and normalized by the sum of all the expected substitutions for the same category.

### Estimation of GC content

Current and ancestral GC content were estimated for 0- and 4-fold degenerate sites as proxies for non-synonymous and synonymous GC content, respectively. Current GC content was defined as the sum of G and C nucleotides in the respective lineage. As described above, ancestral GC content refers to the GC content at the most recent internal node of a particular tip of the tree. In addition, we computed equilibrium GC content for non-synonymous and synonymous changes (GC*) as W-to-S/(W-to-S + S-to-W) substitution rates separately for each lineage. Lineage-specific ΔGC was estimated as the difference between lineage-specific GC* and the lineage-specific ancestral GC content.

### Life-history traits

Estimates of body mass (grams), maximum longevity (years), and age of sexual maturity (days) were retrieved from Figuet et al. [30]*.* Briefly, the authors retrieved body mass estimates from the CRC Handbook of Avian Body Masses [72], while longevity and age of sexual maturity were retrieved from the literature (for detailed information see supplementary tables of the original publication [30]). All estimates were log-transformed to the base of 10.

### Statistical analyses

All statistical analyses were performed in R v. 3.2.2 (R core team 2015). Pearson’s correlation coefficient was used to test for correlations among variables. To account for phylogenetic relationships of species in correlation analysis, we used the ape package in R in order to compute phylogenetically independent contrasts following the method described by Felsenstein [73].

## Additional files

Additional file 1: **Table S1.** Species specific dN/dS estimates for different substitution categories. (XLSB 56 kb)

Additional file 2: Supplementary Tables S2-S6, and Supplementary Figures S1-S8. (DOCX 608 kb)
