# Supplementary material for: GC-biased gene conversion conceals the prediction of the nearly neutral theory in avian genomes
Source: Genome Biol. 2019 Jan 7;20:5. doi: 10.1186/s13059-018-1613-z (PMC6322265; doi:10.1186/s13059-018-1613-z)
Supplement: Supplementary file 2 — Supplementary Tables S2-S6, and Supplementary Figures S1-S8. (DOCX 608 kb) [file 13059_2018_1613_MOESM2_ESM.docx]

**Additional file 2**

**Supplementary tables**

To address the potential effect of phylogenetic relationships in the LHTs we used the ape package in R to compute phylogenetically independent contrasts using the method described by Felsenstein (1985). This method accounts for the phylogenetic relationships of species when estimating the correlation between *d_N_/d_S_* and LHTs.

Table S2: Pearson correlation coefficients (*R*) and their statistical significance between *d_N_/d_S_* for different substitution categories and different proxies for *N_e_* with correction for phylogenetic relationships of species. Significant correlations are highlighted in bold.

| Life history trait | Total | | GC-conservative | | S-to-W | | W-to-S | |
| --- | --- | --- | --- | --- | --- | --- | --- | --- |
|  | *R* | *p*-value | *R* | *p*-value | *R* | *p*-value | *R* | *p*-value |
| Body mass | 0.28 | 7.24e-02 | **0.60** | **9.12e-06** | -0.09 | 5.52-e01 | **0.36** | **1.52-e02** |
| Longevity | 0.09 | 5.70e-01 | 0.24 | 1.41e-01 | -0.12 | 4.51e-01 | 0.20 | 2.22e-01 |
| Age of sexual maturity | 0.01 | 9.65e-01 | 0.29 | 5.96e-01 | -0.19 | 2.25e-01 | 0.16 | 3.06e-01 |

Table S3: Pearson correlation coefficients (*R*) and their statistical significance between *d_N_/d_S_* for different substitution categories using a gene-by-gene approach and different proxies for *N_e_* without correction for phylogenetic relationships of species. Significant correlations are highlighted in bold.

| Life history trait | Total | | GC-conservative | | S-to-W | | W-to-S | |
| --- | --- | --- | --- | --- | --- | --- | --- | --- |
|  | *R* | *p*-value | *R* | *p*-value | *R* | *p*-value | *R* | *p*-value |
| Body mass | -0.15 | 3.06e-01 | **0.55** | **6.42e-05** | **-0.33** | **2.19-e02** | -0.01 | 5.07-e01 |
| Longevity | -0.07 | 6.87e-01 | **0.35** | **2.79e-02** | -0.26 | 1.03e-01 | -0.05 | 7.42e-01 |
| Age of sexual maturity | -0.14 | 3.45e-01 | **0.52** | **2.81e-02** | -0.19 | 2.15e-01 | 0.03 | 8.23e-01 |

Table S4: Pearson correlation coefficients (*R*) and their statistical significance between *d_N_/d_S_* for different substitution categories using a gene-by-gene approach and different proxies for *N_e_* with correction for phylogenetic relationships of species. Significant correlations are highlighted in bold.

| Life history trait | Total | | GC-conservative | | S-to-W | | W-to-S | |
| --- | --- | --- | --- | --- | --- | --- | --- | --- |
|  | *R* | *p*-value | *R* | *p*-value | *R* | *p*-value | *R* | *p*-value |
| Body mass | -0.11 | 3.07e-01 | **0.58** | **1.85e-05** | -0.24 | 1.03-e01 | -0.01 | 9.26-e01 |
| Longevity | -0.12 | 4.69e-01 | 0.28 | 8.62e-02 | -0.20 | 2.12e-01 | -0.09 | 5.71e-01 |
| Age of sexual maturity | -0.26 | 9.39e-01 | **0.36** | **1.62-02** | -0.23 | 1.31e-01 | -0.12 | 3.55e-01 |

Table S5: Pearson correlation coefficients and their statistical significance between *d_N_/d_S_* for different substitution categories and body mass for genes with low and high GC content, and for genes located in microchromosomes or macrochromosomes with correction for phylogenetic relationships of species. Significant correlations are highlighted in bold.

|  | Total | | GC-conservative | | S-to-W | | W-to-S | |
| --- | --- | --- | --- | --- | --- | --- | --- | --- |
|  | *R* | *p*-value | *R* | *p*-value | *R* | *p*-value | *R* | *p*-value |
| GC High | 0.12 | 4.21-01 | **0.43** | **2.45e-03** | -0.04 | 7.87e-01 | 0.22 | 1.34e-1 |
| GC Low | **0.35** | **1.65e-02** | **0.61** | **5.88e-06** | 0.03 | 8.28e-01 | **0.37** | **1.06e-02** |
| Micro  chromosomes | 0.23 | 1.09e-01 | **0.51** | **3.17e-04** | -0.09 | 5.46-01 | **0.37** | **1.14-e-02** |
| Macro  chromosomes | 0.25 | 8.8e-02 | **0.58** | **2.44e-05** | 0.07 | 6.55e-01 | 0.20 | 1.73e-01 |

Table S6: Pearson correlation coefficients (*R*) and their statistical significance between *d_N_/d_S_* and body mass for different datasets analysed in the present study with correction for phylogenetic relationships of species. Significant correlations are highlighted in bold.

| Dataset | *Neornithes* | | *Noeognites* | | *Neornithes*  GC-conservative | | N*eognithes*  GC-conservative | |
| --- | --- | --- | --- | --- | --- | --- | --- | --- |
|  | *R* | *p*-value | *R* | *p*-value | *R* | *p*-value | *R* | *p*-value |
| Figuet+HMMclean | **0.31** | **4.24e-02** | **0.33** | **4.20e-2** | **0.59** | **3.02e-05** | **0.65** | **6.08e-06** |
| Botero-Castro | **0.54** | **1.67e-04** | **0.61** | **3.78e-05** | **0.67** | **7.10e-07** | **0.75** | **3.08e-08** |
| Original dataset of our study | 0.28 | 7.24e-02 | 0.26 | 8.98e-01 | **0.60** | **9.12e-06** | **0.64** | **3.63e-06** |

**Supplementary figures**

Figure S1. Relationship between *d_N_/d_S_* and body mass for different substitution categories based on the T92X3 model. A) All substitution categories together (Total; black circles), B) GC-conservative substitutions (grey diamonds), C) S-to-W substitutions (blue triangles) and D) W-to-S substitutions (red squares). Each dot corresponds to a terminal branch of the avian phylogeny. Regression lines for each substitution category are shown using the same colour scheme. Pearson correlation coefficients and their statistical significance are provided in the right upper corner of each panel.

Figure S2. Relationship between *d_N_/d_S_* and body mass for different substitution categories based on the L95X3 model. A) All substitution categories together (Total; black circles), B) GC-conservative substitutions (grey diamonds), C) S-to-W substitutions (blue triangles) and D) W-to-S substitutions (red squares). Each dot corresponds to a terminal branch of the avian phylogeny. Regression lines for each substitution category are shown using the same colour scheme. Pearson correlation coefficients and their statistical significance are provided in the right upper corner of each panel.

Figure S3. Relationship between *d_N_/d_S_* and body mass for different substitution categories. A) All substitution categories together (Total; black circles), B) GC-conservative substitutions (grey diamonds), C) S-to-W substitutions (blue triangles) and D) W-to-S substitutions (red squares). Each dot corresponds to a terminal branch of the avian phylogeny. Regression lines for each substitution category are shown using the same colour scheme. Pearson correlation coefficients and their statistical significance are provided in the right upper corner of each panel. Note that this figure is identical to Figure 1 of the main text, except that Neoaves are indicated by filled shapes, and Paleognathae and Galloanserae by hollow shapes.

Figure S4. Relationship between *d_N_/d_S_* and body mass for different substitution categories for the Figuet+HMMclean dataset (1,077 genes). A) All substitution categories together (Total; black circles), B) GC-conservative substitutions (grey diamonds), C) S-to-W substitutions (blue triangles) and D) W-to-S substitutions (red squares). Each dot corresponds to a terminal branch of the avian phylogeny. Regression lines for each substitution category are shown using the same colour scheme. Pearson correlation coefficients and their statistical significance are provided in the right upper corner of each panel. Neoaves are indicated by filled shapes, and Paleognathae and Galloanserae by hollow shapes.

Figure S5. Relationship between *d_N_/d_S_* and body mass for different substitution categories for the Botero-Castro dataset (2,322 genes). A) All substitution categories together (Total; black circles), B) GC-conservative substitutions (grey diamonds), C) S-to-W substitutions (blue triangles) and D) W-to-S substitutions (red squares). Each dot corresponds to a terminal branch of the avian phylogeny. Regression lines for each substitution category are shown using the same colour scheme. Pearson correlation coefficients and their statistical significance are provided in the right upper corner of each panel. Neoaves are indicated by filled shapes, and Paleognathae and Galloanserae by hollow shapes.

Figure S6. Boxplots for relative difference in non-synonymous and synonymous substitution rates between GC-rich and GC-poor genes, estimated as: ((GC-rich rate - GC-poor rate)/ branch length). The vertical dashed line indicates a relative difference of 0. *p*-values for a *t*-test comparing each distribution to 0 are provided.

 Figure S7: Relationships between *d_N_/d_S_* and body mass separately for genes located on macrochromosomes and microchromosomes. Macrochromosomes (filled shapes) and microchromosomes (hollow shapes). A) All substitution categories together (Total; black circles), B) GC-conservative substitutions (grey diamonds), C) S-to-W substitutions (blue triangles) and D) W-to-S substitutions (red squares). Each dot corresponds to a terminal branch of the avian phylogeny. Regression lines for each substitution category are shown using the same colour scheme. Solid lines correspond to GC-poor genes, dashed lines to GC-rich genes.

Figure S8. Boxplots for relative difference in non-synonymous and synonymous substitution rates between genes located on macrochromosomes and microchromosomes, respectively. The vertical dashed line indicates a relative difference of 0. *p*-values for a *t*-test comparing each distribution to 0 are provided.
